# Reliability and validity of a vertical numerical rating scale supplemented with a faces rating scale in measuring fatigue after stroke

**DOI:** 10.1186/s12955-015-0290-9

**Published:** 2015-06-30

**Authors:** Li-ling Chuang, Keh-chung Lin, An-lun Hsu, Ching-yi Wu, Ku-chou Chang, Yen-chen Li, You-lin Chen

**Affiliations:** Department of Physical Therapy and Graduate Institute of Rehabilitation Science, College of Medicine, Chang Gung University, Taoyuan, Taiwan; Healthy Ageing Research Center, Chang Gung University, 259 Wen-hua 1st Rd., Guishan, Taoyuan, Taiwan; School of Occupational Therapy, College of Medicine, National Taiwan University, Taipei, Taiwan; Division of Occupational Therapy, Department of Physical Medicine and Rehabilitation, National Taiwan University Hospital, Taipei, Taiwan; Department of Physical Therapy, Mackay Memorial Hospital, Taipei, Taiwan; Department of Occupational Therapy and Graduate Institute of Behavioral Sciences, College of Medicine, Chang Gung University, Taoyuan, Taiwan; Department of Neurology, Chang Gung Memorial Hospital, Kaohsiung, Taiwan; Department of Physical Medicine and Rehabilitation, Physical Therapy, Chang Gung Memorial Hospital, Taoyuan, Taiwan

**Keywords:** Fatigue, Reliability, Numerical rating scale, Faces rating scale, Stroke

## Abstract

**Background:**

Poststroke fatigue is a persistent and distressing symptom among stroke survivors. In this study, we investigated the reliability and validity of a vertical numerical rating scale supplemented with a faces rating scale (NRS-FRS) in measuring poststroke fatigue.

**Methods:**

The fatigue intensity of 106 individuals with stroke was measured twice, 1 week apart, using a vertical NRS-FRS to measure test-retest reliability. The intraclass correlation coefficient, a relative reliability index, was calculated to examine the degree of consistency and agreement between the two test occasions. Absolute reliability indices, including the standard error of measurement, minimal detectable change, and Bland-Altman limits of agreement, were used to quantify measurement errors and determine systematic biases of the two test occasions. We also administered the vertical NRS concurrently as a comparator measure for assessing fatigue in 50 consecutive patients with stroke who were recruited later in the study period. The Spearman rank correlation coefficient (*ρ*) was used to examine the concurrent validity of the NRS-FRS. Discriminant validity was assessed by means of receiver operating characteristic curves, sensitivity, and specificity.

**Results:**

The intraclass correlation coefficient was 0.95 for the NRS-FRS. The standard error of measurement and the minimal detectable change at the 95 % confidence interval of the NRS-FRS were 0.50 and 1.39, respectively. The Bland-Altman analyses showed no significant systematic bias between the repeated measurements. A narrow range of the limits of agreement was shown on the Bland-Altman plot, indicating the NRS-FRS had high stability and low variation between the two test occasions. The correlations between the NRS-FRS and NRS were good at test (*ρ* = 0.85) and retest (*ρ* = 0.84). Compared with the NRS cutoff value of ≥1, sensitivity with the NRS-FRS at test and retest was 94 and 92 % and specificity was 79 and 90 %, respectively.

**Conclusions:**

This study provides further evidence of the reliability and validity of the NRS-FRS in measuring fatigue intensity in patients with stroke. The NRS-FRS had high sensitivity and specificity. The NRS-FRS may be a reliable and valid measure for clinicians and researchers to assess fatigue and determine whether a real change has occurred in groups and at the individual level of patients with stroke.

## Introduction

Poststroke fatigue is a persistent and distressing symptom among stroke survivors [1, 2], with a prevalence ranging from 38 to 77 % [3, 4]. Fatigue often impedes rehabilitation [5] and has serious effects on a sense of not being in control [6], a higher risk of suicide [7], increased mortality [3], increased energy cost for gait deficits [8], and reduced physical fitness [9]. The recognition of poststroke fatigue has driven the need of researchers and clinicians for a valid, reproducible, and feasible measurement for the screening and diagnosis of fatigue.

Fatigue after stroke is a multifaceted phenomenon associated with demographic, physiological, psychocognitive, and organic factors [2, 5, 10]. The multidimensional nature of fatigue creates difficulties for clinicians and researchers in assessing the patient’s condition and implementing the best treatment. Fatigue consists of acute and chronic fatigue [9, 11, 12]. Acute fatigue is perceived as fatigability (exertional fatigue), which develops after certain activity, can be alleviated by rest, and is associated with neurologic impairment. Alternatively, chronic fatigue is a state of weariness, which is unrelated to activity or exertion, cannot be relieved after rest, and is associated with prolonged stress or illness. Thus, poststroke fatigue has been defined as having components of physical, cognitive, and social fatigue, which may vary by individuals [2]. Although poststroke fatigue has mental and psychological aspects as well as a physical basis [1], physical fatigue is much greater influence in patients’ experience of fatigue and interfered with their daily activities [13].

Measuring fatigue remains an ongoing challenge for clinical trials of fatigue management in stroke, because no gold standard measure is available for poststroke fatigue [3, 14–16]. Poststroke fatigue has been viewed as difficult to measure adequately and is thus neglected as a consequence [1, 12]. The assessment of fatigue must consider the feasibility of a measure and individual’s ability to successfully complete the measurement, which may depend on the severity of fatigue and cognitive, visual, or language deficiencies of stoke patients. The measurement for fatigue should be feasible with regard to being simple to administer, easy to score, and completed with minimal time and effort, for not adding fatigue intensity [17]. In this regard, single-item measure of fatigue intensity, such as the visual analog scale (VAS) [9, 12, 17, 18] and numerical rating scale (NRS) [19, 20], seem to be more advantageous than multiple-item measures [21]. For people experiencing severe fatigue, multidimensional fatigue measures may increase the burden of responding [22]. Stroke patients may have problems recalling their fatigue levels of prior week, which might affect the accuracy of the data [11]. Some people with right hemispheric stroke might present hemineglect to their left side [23]; other people with left hemispheric stroke might have difficulty in fully understanding an instruction [24]. Yet, the VAS is not recommended for geriatric patients [25] or stroke patients with cognitive or visuospatial impairments [23].

Alternatively, the NRS is commonly used to estimate fatigue intensity in individuals with stroke [19], multiple sclerosis [21], spinal cord injury [26], fibromyalgia [27], and cancer [28]. Previous studies have demonstrated that the NRS is a valid, reliable, and highly responsive measure of fatigue in patients with rheumatoid arthritis [29, 30] and multiple sclerosis [21]. The NRS evaluates patient’s fatigue level at a 0-to-10 scale. The chosen number signifies the severity of subject’s fatigue, with 0 indicating no fatigue and 10 indicating the worst possible fatigue. The NRS is extremely easy to administer and has shown good sensitivity, clinical relevance, and usefulness [19, 28, 29]. Therefore, the NRS is a suitable instrument for stroke patients because of validity, reliability, and preference. However, in consideration of possible lacking cognitive and visuospatial functions in stroke patients or elderly participants, an adaptation of the NRS to assess fatigue may be needed.

The faces rating scale (FRS) has been successful in measuring pain in cognitively impaired patients [24] and in illiterate patients [31–33]. The 0–10 vertical NRS supplemented with the Wong-Baker FRS was reliable in measuring pain after stroke [34]. Therefore, a vertical NRS scale incorporating with the FRS would be an a priori preferable measurement of poststroke fatigue. Hence, the 6-face Wong-Baker FRS was used to make it comparable with the NRS in scoring by use of a common metric (0–10) in the present study. Our aim was to determine the test-retest reliability and validity of the vertical NRS incorporated with the FRS (NRS-FRS) for assessing fatigue in people with stroke.

## Methods

### Participants

Stroke patients who were diagnosed between December 2013 and January 2015 were recruited at three medical centers. The inclusion criteria were (*a*) a first-ever stroke onset of at least 3 months before recruitment, (*b*) enrollment in an outpatient rehabilitation program, (*c*) ability to follow study instructions and complete the scale (Mini-Mental State Examination score of ≥22), and (*d*) no participation in experimental rehabilitation or drug studies during the study period. The local Institutional Review Board of Mackay Memorial Hospital and Chang Gung Memorial Hospital approved the study procedures, and all participants provided written informed consent.

The exclusion criteria were (*a*) physician-determined major medical problems, (*b*) inability to complete questionnaires or study outcome measures because of severe cognitive impairment, neglect, or attention deficits, and (*c*) irregular use of medications for fatigue or other fatigue-relieving treatment during the study period.

### Procedure

Eligible patients who received outpatient rehabilitation were invited to participate. For determining test-retest reliability of the scale, NRS-FRS was assessed twice with a 1-week interval to reduce the memory effect of the first assessment, and at the same time of day to minimize diurnal variation in fatigue. Test and retest assessments were administered by the same research assistant. In addition, 50 consecutive participants were asked to indicate the severity of their fatigue successively on the NRS-FRS and the vertical NRS. The vertical NRS was used as a comparator measure to test the concurrent validity of NRS-FRS.

### Outcome measure of fatigue

**Fig. 1:**
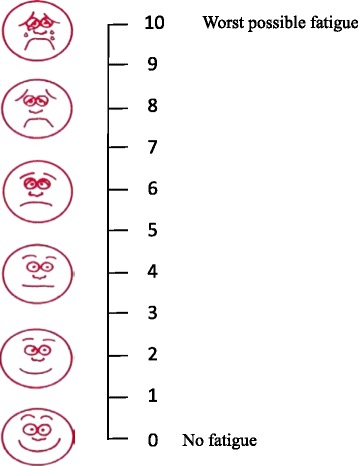
Numerical rating scale supplemented with a faces rating scale for self-reported fatigue intensity

Fatigue was defined as a feeling of physical tiredness and lack of energy [35], as assessed using the NRS-FRS and the vertical NRS. Participants were provided a full explanation of the fatigue measures and received instructions on how to complete the scales. To facilitate scoring the intensity of participants’ fatigue, the NRS-FRS was a combination of the vertical NRS with word anchors on a scale of 0 to10 and the 6 facial expressions of Wong-Baker FRS (Fig. 1).

A 10-cm vertical line anchored by a smiling face with the bottom number 0 to indicate “no fatigue” and a crying face with the top number 10 to indicate “worst possible fatigue.” Participants were asked to point only to a number, not a face, on the NRS-FRS that best represented their present level of fatigue (“How fatigued do you currently feel?”) using the 10-point single-item fatigue scale (0 = “no fatigue” and 10 = “worst possible fatigue”). Fatigue severity units included none, 0; mild, 1 to 3; moderate, 4 to 6; and severe, 7 to 10, giving it ordinal properties of measurement [29]. The higher the NRS-FRS score, the higher the fatigue. The 10-point fatigue scale is well validated to assess fatigue in people with cancer [28].

### Data analysis

The relative reliability of the NRS-FRS was determined through intraclass correlation coefficient (ICC) using a 2-way mixed-effect model with an agreement coefficient [36]. ICCs that exceed 0.75 indicate good reliability [37]. We used the standard error of measurement (SEM), the minimal detectable change (MDC), and Bland-Altman analyses to quantify the absolute reliability.

The SEM indicates within-subject variability in repeated measures for a group of individuals [38]. The MDC_95_ is the smallest change necessary to exceed the measurement error of repeated measures that indicates a real change at the 95 % confidence interval (CI) level for a single individual [38, 39]. Bland-Altman analyses were used to indicate systematic bias between repeated measurements [40]. The Bland-Altman plot illustrates the agreement between the two test occasions (time 1 and time 2) and identifies possible outliers. The 95 % CI of the mean difference was used to determine systematic bias. If zero is included within the 95 % CI, no significant systematic bias between measurements can be inferred [40]. The 95 % limits of agreement (LOA) were used to examine the natural variation over time, with a narrow LOA indicating higher stability [41].

We studied concurrent validity to validate the NRS-FRS with the NRS obtained concurrently in a subsample of participants at 2 study visits [37]. The Spearman rank correlation coefficient (*ρ*) was used to examine the relation between the NRS-FRS and NRS at test and retest. We used the following criteria to interpret the magnitude of the correlation coefficients: <0.25 indicating low correlations, 0.25 to 0.5 indicating fair correlations, 0.5 to 0.75 indicating moderate-to-good correlations, and >0.75 indicating good-to-excellent correlations [37]. Discriminant validity was assessed by means of receiver operating characteristic (ROC) curves, sensitivity, and specificity. ROC analysis was used to define the best NRS-FRS cutoff score of the 50 participants. ROC curves were plotted to determine the area under the curve (AUC), which represents the ability of the NRS-FRS to discriminate between those with and without fatigue [37]. The sensitivity and specificity of the NRS-FRS were calculated using the cutoff point ≥1 and were represented by ROC curves.

## Results

The 106 participants were a mean age of 53.63 years, and the average time after stroke onset was 24.40 months (Table 1). The detailed characteristics of the participants and the descriptive statistics for the NRS-FRS in the two test occasions are reported in Table 1.

**Table 1:** Characteristics of the Participants (*n* = 106)

Characteristic	No (%), mean (SD), or median (range)
Sex	
Male	77 (72.6 %)
Female	29 (27.4 %)
Age, year	53.63 (11.25)
Localization	
Right hemisphere	48 (45.3 %)
Left hemisphere	58 (54.7 %)
Interval after stroke onset, months	24.40 (24.11)
Brunnstrom stage of upper limb	
Proximal part	4 (1–6)
Distal part	3 (1–6)
Brunnstrom stage of lower limb	4 (3–5)
Fugl-Meyer Assessment of upper limb	33.74 (17.62)
Fugl-Meyer Assessment of lower limb	21.18 (6.94)
First assessment of fatigue intensity with NRS-FRS	1.93 (2.30)
Patients with severe fatigue of 7–10	4 (3.8 %)
Patients with moderate fatigue of 4–6	20 (18.9 %)
Patients with mild fatigue of 1–3	40 (37.7 %)
Patients with no fatigue	42 (39.6 %)
Second assessment of fatigue intensity with NRS-FRS	1.77 (2.21)
Patients with severe fatigue of 7–10	4 (3.8 %)
Patients with moderate fatigue of 4–6	16 (15.1 %)
Patients with mild fatigue of 1–3	42 (39.6 %)
Patients with no fatigue	44 (41.5 %)
Mini Mental State Exam scores	27.56 (2.43)

*Abbreviation: SD* standard deviation, *NRS-FRS* numerical rating scale supplemented with faces rating scale

**Fig. 2:**
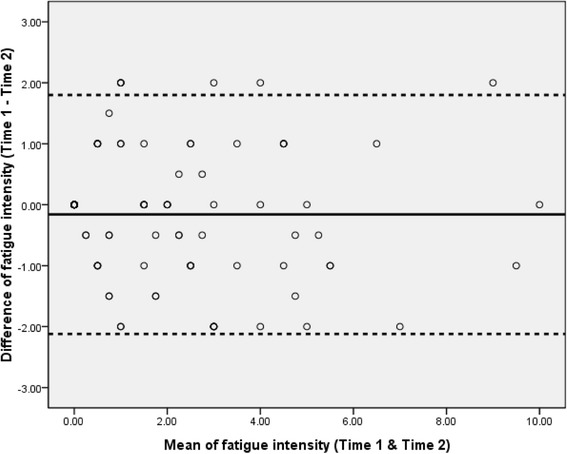
Bland-Altman plot for the test-retest reliability. The plot illustrates the agreement between time 1 and time 2 and identifies possible outliers. Each sample is represented on the graph by conveying the mean value of the 2 assessments (*x*-axis) and the difference between the 2 assessments (*y*-axis). The mean difference was the estimated bias, and the standard deviation (SD) of the differences measured the fluctuations around this mean (outliers being above 1.96 SD_diff_). Reference lines shows mean difference between time 1 and time 2 (*solid line*), and 95 % limits of agreement for the mean difference (*broken lines*)

As detailed in Table 2, the ICC for the NRS-FRS was 0.95 (95 % CI, 0.92–0.96), indicating good relative reliability of the NRS-FRS. The SEM and MDC_95_ of the NRS-FRS were 0.50 and 1.39, respectively. The mean difference between the test-retest measures of the NRS-FRS was close to 0 (−0.16). The 95 % CI for the mean difference included 0 (−0.36 to 0.04), demonstrating that there was no significant systematic bias between test-retest measures in poststroke fatigue. The Bland-Altman plot (Fig. 2) that was representative of the NRS-FRS showed the variability between the test-retest measures. The repeatability for most of the test-retest measures was within the 95 % CI. The LOA range was −2.12 to 1.80, and 4 outliers are shown on the plot.

**Table 2:** Relative and absolute reliabilities of a numerical rating scale supplemented with a faces rating scale

Scale	ICC (95 % CI)	SEM	MDC_95_	Bland-Altman analysis				
				d	SD_diff_	SE of d	95 % CI of the d	LOA
NRS-FRS	0.95 (0.92–0.96)	0.50	1.39	−0.16	1.00	0.10	−0.36 to 0.04	−2.12 to 1.80

*NRS-FRS* numerical rating scale supplemented with faces rating scale, *ICC* intraclass correlation coefficient, *CI* confidence interval, *SEM* standard error of measurement = SD_pooled_ × √(1 − ICC)], where *SD*_*pooled*_ is the standard deviation for all observations from test occasions 1 and 2, *MDC*_*95*_ minimal detectable change at the 95 % CI level = 1.96 × √2 × SEM = 1.96 × √2 × SD_pooled_ × √(1 − ICC)], where *1.96* is the 2-tailed tabled *z* value for the 95 % CI and *√2* represents the variance of 2 measures, *d* mean of difference between the two test sessions (test session 2 minus test session 1), *SD*_*diff*_ standard deviation of mean difference, *SE* standard error, *95 % CI of the d* mean difference ± 1.96 × SE = d ± 1.96 × (SD_diff_ /√*n*), where *n* is the sample size, *95 % LOA* 95 % limits of agreement = d ± 1.96 SD_diff_

**Fig. 3:**
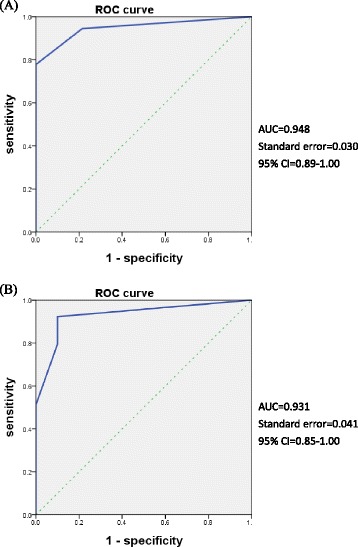
Receiver operating characteristic (ROC) curves of the numerical rating scale-faces rating scale (NRS-FRS) at (**a**) test and (**b**) retest (NRS cutoff point of 1 as a criterion measure). The area under the curve (AUC) was 0.948 for test and 0.931 for retest

The correlations between the NRS-FRS and NRS were good at test (*ρ* = 0.85) and retest (*ρ* = 0.84), as reported in Table 3. Compared with the criterion measure of the NRS, the sensitivity of NRS-FRS ≥1 for fatigued patients at test and retest was 94 and 92 % and specificity was 79 and 90 %, respectively (Table 4). ROC curves of fatigued (NRS ≥1) and not fatigued (NRS <1) with the NRS-FRS at test and retest are shown in Fig. 3. The AUC was 0.948 (95 % CI, 0.89–1.00) for test and 0.931 (95 % CI, 0.85–1.00) for retest.

**Table 3:** Concurrent validity (Spearman Rank Correlation Coefficient) of the NRS-FRS and NRS at test and retest

Criterion measure	Test	Retest
	NRS-FRS (95 % CI)	NRS-FRS (95 % CI)
NRS	0.85* (0.75–0.91)	0.84* (0.73–0.91)

*P < 0.01

**Table 4:** Sensitivity and Specificity of the NRS-FRS at Test and Retest

Criterion measure	Cut-off score	Test NRS-FRS			Retest NRS-FRS		
		Sensitivity, %	Specificity, %	AUC	Sensitivity, %	Specificity, %	AUC
NRS	1	94	79	0.948	92	90	0.931
	2	97	79	0.913	85	75	0.900
	3	96	75	0.907	77	89	0.910
	4	94	82	0.921	73	79	0.859
	5	89	88	0.959	56	90	0.871

*AUC* Area under the curve

## Discussion

This study provides evidence of the test-retest reliability and validity of the vertical NRS-FRS in quantifying the intensity of fatigue in individuals with stroke. The relative and absolute reliability of the NRS-FRS showed good test-retest reliability, with high agreement, small measurement error, and no systematic bias for the assessment of poststroke fatigue. The concurrent validity of the NRS-FRS was good. The sensitivity and specificity of the NRS-FRS were high. These findings suggest that the vertical NRS-FRS may be a reliable and valid instrument to assess poststroke fatigue. Moreover, measuring fatigue provides additional information that is essential to understand disease outcome from the patient’s perspective.

Limited empirical evidence about the reliability of instruments for measuring poststroke fatigue has seriously hampered efforts to synthesize common knowledge about fatigue. Establishing the reliability of a tool for the adequate assessment of fatigue is an important prerequisite before the tool is adopted as a standard measure of poststroke fatigue. Test-retest reliability is the ability of an outcome measure to capture similar scores on 2 separate occasions of test administration, given that the patient’s condition has not changed [37]. The ICC gives a measure of consistency or agreement of values within cases [42]. The ICC value in this study (0.95) indicated a high degree of agreement between the test-retest measures and good reproducibility of the vertical NRS-FRS. Our data were in line with values reported in previous studies, which indicated good reliability of the NRS in patients with multiple sclerosis (ICC = 0.97) [21] and rheumatoid arthritis (ICC = 0.79) [29].

Determination of the absolute reliability of measures is critical to ensure repeated measurements with satisfactory stability and sensitivity to real changes over time [43]. Reliable outcome measures demonstrate small measurement errors for a group of patients and small true changes for an individual patient. The SEM and MDC_95_ of the test-retest measures provide the absolute values of the measurement errors between repeated measures and determine whether changes in repeated measures for a group and for an individual are real, respectively [44, 45]. From the result of the MDC_95_ of the NRS-FRS, if the change in repeated measures of the NRS-FRS for a stroke patient was more than 1.39, then the change was interpreted as a real change or a true change beyond measurement error at the 95 % CI. The SEM and MDC_95_ of the NRS-FRS were similar to those in a previous study of the Fatigue Severity Scale (FSS) for measuring fatigue in polio survivors (SEM = 0.56, MDC_95_ = 1.55) [46] despite different patient populations and outcome measures.

Limited studies have presented the test-retest reliability of the fatigue scales by the Bland-Altman method [16, 29]. In the present study, the Bland-Altman statistics for the NRS-FRS in individuals with stroke indicated no significant systematic bias and narrow LOA between the repeated measures. These results were similar to the use of the NRS in measuring fatigue in rheumatoid arthritis [29], the Fatigue Assessment Scale in evaluating fatigue after stroke [16], and the NRS-FRS in measuring pain after stroke [34]. The Bland-Altman plots for test-retest reliability of the NRS in rheumatoid arthritis demonstrated small differences on repeated measurement and no bias in the distribution [29]. Test-retest agreement for the Fatigue Assessment Scale in stroke individuals had the narrowest LOA, and the mean difference between test and retest measurements was not significant [16]. Generally, this study found that the absolute reliability of the NRS-FRS in assessing poststroke fatigue is good, with no bias in the distribution and small differences on repeated measurement. The mean difference between the two testing occasions was close to zero, and the 95 % CI of the mean difference included zero. From the Bland-Altman plot, the narrow range of the LOA and the 4 outliers in the NRS-FRS indicated a high stability and less natural variation over time.

The diagnostic value of the NRS-FRS to assess fatigue in stroke patients was also analyzed by comparing to NRS. The correlations between the NRS-FRS and NRS is high, fluctuating only slightly at test (*ρ* = 0.85) and retest (*ρ* = 0.84). This suggested that the relationships between the tests are relatively stable over a 1-week interval, which reflects constant and true relationships between the tests and indicates that both measure the same construct. The NRS has been validated for assessing fatigue severity in patients with ankylosing spondylitis [47] and rheumatoid arthritis [30]. Despite the differences in sample characteristics, the findings of our study are consistent with results of prior research in supporting the NRS and NRS-FRS as being valid measures for fatigue intensity. When the NRS-FRS is used to distinguish fatigue from no fatigue, the area under the ROC curve is very high at test (AUC = 0.948) and retest AUC = 0.931). These results highly suggest that the NRS-FRS is an appropriate tool for the assessment of physical fatigue in stroke patients.

Despite high correlation and AUC, higher cut-off values of the NRS-FRS might be insufficiently accurate to guide fatigue management. For example, when NRS-FRS cutoff value was set ≥1 for detecting fatigue at the first assessment, 6 % of the patients with no fatigue would be incorrectly classified as having fatigue, and 21 % of patients with fatigue would be incorrectly classified as no fatigue. If the cutoff point was increased to >5, 11 % of stroke patients with no fatigue would be incorrectly classified as having fatigue, and 12 % with fatigue would be classified as having no fatigue.

Most fatigue studies in stroke rely on questionnaires, such as FSS [10, 48, 49], Multidimensional Fatigue Symptom Inventory (MFSI) [16], VAS [9, 12, 17, 18], and NRS [19, 20]. The FSS and MFSI are validated measures for fatigue, but both rely on retrospective recall of fatigue during the preceding 1 week rather than a real-time assessment. In contrast, VAS and NRS are single-item measures of self-reported fatigue severity that prospectively capture real-time fatigue. Thus, VAS and NRS did not have recall bias and respondent burden is low [21]. Actually, the VAS has been shown to be a reliable and valid instrument for the quantitative assessment of fatigue in healthy subjects [17], patients with sleep disorders [17], and people with chronic stroke [12]. However, the VAS is influenced by eye-hand coordination problems [11]. Patients with paralysis, tremors, or visual impairment are unable to complete the VAS reliably [17]. The application of VAS is limited to the motor, cognitive, and visual abilities of the subjects.

Patients with poststroke fatigue may have problems completing long questionnaires. The feasibility of a fatigue scale is frequently the element that determines the initial choice of an instrument for individuals with stroke. It should be short, easy to understand and answer, have a minimal respondent burden, and be reliable when replicated [17]. Fatigue intensity is probably the easiest and simplest dimension of fatigue to assess and a reasonable way to begin the discussion about fatigue. All participants in this study were able to complete the NRS-FRS. The reliability and validity results of this study showed that a simple approach of combining the NRS with the FRS created a reliable and valid tool for assessing poststroke fatigue. The NRS-FRS is easy to understand, quick to complete, simple to score, and does not place an excessive burden on patients. In the absence of fully validated gold standards, the vertical NRS-FRS, with good test-retest reliability and validity, could be used to monitor real-time fatigue, facilitate faster communication between patients and clinicians regarding their fatigue experience and response to treatment, and allow for future comparability across different studies. Since fatigue levels may fluctuate throughout the day [21] and exertional fatigue may be perceived after activities [9], it is important to administer the NRS-FRS at the same time of day and to avoid administering it after physical and cognitive activity.

However, we acknowledge that the one-dimensional measurement may have value as a screening tool for documentation but not fully interpret the intricacies of the symptom and may not address the linkage between fatigue intensity and functional limitations [9, 26]. Poststroke fatigue was predominantly physical rather than mental [13, 35]. Chalder et al. recommended that fatigue severity be accompanied by an assessment of fatigue interference with activities, which may offer a more thorough description of the fatigue experience, capture the most salient issues, and trigger a comprehensive list of problems [50]. In future studies, we propose a positive screening of the NRS-FRS should be followed up with a more comprehensive assessment of patients’ perceptions of functional impairment and interference due to fatigue, in addition to single-symptom questions measuring fatigue intensity, to facilitate a more complete description of the fatigue experience in daily life for individuals with stroke.

A good example of a more comprehensive instrument is the Brief Fatigue Inventory (BFI), which was developed to assess the severity of fatigue and the effect of fatigue on daily functioning in the past 24 h for patients with cancer [51]. The BFI has 9 items, and each item is rated on an 11-point NRS. The BFI might be an optimal outcome measurement between multidimensional fatigue measures and a single-item measurement to reveal a tremendous amount about an individual’s fatigue status. Future study might consider investigating the psychometric properties of the BFI in individuals with stroke.

Some limitations of our study warrant consideration. First, all participants in the present study completed the NRS-FRS at two assessments, with a 1-week interval, at the same time of day to minimize any diurnal variation in fatigue. Fatigue was measured as a single time-point assessment; that is, current fatigue intensity, which might not reflect overall fatigue on the testing day. Future studies may consider measuring fatigue at different times of the day to facilitate a better understanding of daily fluctuations in poststroke fatigue and to improve the psychometric properties of the NRS-FRS.

Second, future studies need to identify predictors of poststroke fatigue to address fatigue issues with an intervention in people with stroke. To explore the effectiveness of an intervention to manage fatigue or the progression of fatigue, the ability of the NRS-FRS to detect change over time requires further development.

In conclusion, our research shows that the vertical NRS-FRS has good test-retest reliability and validity in measuring physical fatigue after stroke, with good agreement, low measurement error, and high sensitivity and specificity.

## Abbreviations

FSS: Fatigue Severity Scale
MFSI: Multidimensional Fatigue Symptom Inventory
VAS: Visual Analog Scale
NRS: Numerical Rating Scale
FRS: Faces Rating Scale
NRS-FRS: NRS incorporated with the FRS
ICC: Intraclass correlation coefficient
SEM: Standard error of measurement
MDC: Minimal detectable change
LOA: Limits of agreement
ROC: Receiver operating characteristic
AUC: Area under the curve
